# High-concentrated platelet-rich plasma (PRP) versus placebo in osteoarthritis in the thumb base: study protocol for an assessor-blinded randomized controlled trial

**DOI:** 10.1186/s13063-024-08636-2

**Published:** 2024-11-26

**Authors:** Johanna von Kieseritzky, Maria Wilcke

**Affiliations:** 1 Department of Clinical Science and Education, Karolinska Institutet, Stockholm, Sweden; 2 Department of Hand Surgery, Södersjukhuset, Stockholm, Sweden

**Keywords:** Thumb, Osteoarthritis, Platelet-rich plasma, Randomized controlled trial, Placebo, Carpometacarpal joint

## Abstract

**Background:**

Osteoarthritis in the thumb base (trapeziometacarpal joint, CMC-1 joint) is prevalent, particularly among middle-aged and elderly women, causing significant disability. Conservative treatments, including steroid injections, have been questioned for their efficacy, prompting exploration into alternative therapies such as platelet-rich plasma (PRP) injections. This randomized, double-blinded, controlled trial aims to evaluate the effectiveness of high-concentration PRP (platelet-rich plasma) injection compared to saline (placebo) in reducing pain and disability in patients with thumb base osteoarthritis.

**Methods:**

Patients meeting inclusion criteria will be randomized and blinded, with injections administered under sterile conditions and radiological guidance.

With a planned sample size of 90 patients recruited from the Department of Hand Surgery at Södersjukhuset, Stockholm, the study will assess pain relief and functional improvement at 3, 6, and 12 months post-injection. The primary outcome measure is pain on load (numerical rating scale) at 6 months, with secondary outcomes including patient-reported outcomes, key pinch, grip strength, abduction of the thumb, and time to intervention within 1 year.

Statistical analyses will employ non-parametric tests, chi-square tests, and generalized estimating equations to compare outcomes between the PRP and placebo groups.

**Discussion:**

The study aims to provide evidence regarding the efficacy of high-concentration PRP injections for thumb base osteoarthritis. If PRP proves superior to saline in reducing pain and improving function, it could offer a promising alternative treatment. Conversely, if PRP does not demonstrate significant benefits over placebo, its use for this condition is not justified. This study seeks to address the current gap in evidence regarding the efficacy of PRP injections for thumb base osteoarthritis.

**Trial registration:**

The study has been approved by the Swedish Ethical Review Authority (2023–06860-01 and 2024–01238-02) and is registered on ClinicalTrials.gov (NCT06193499) 2024–01-04.

**Supplementary Information:**

The online version contains supplementary material available at 10.1186/s13063-024-08636-2.

## Background

Osteoarthritis in the thumb base (trapeziometacarpal joint, CMC-1 joint) is very common, especially from middle age and in women [1, 2] and may cause significant disability [3].

Conservative treatment includes patient education in joint protection, splints, exercises, analgesics, and intra-articular steroid injections [4–7]. The effect of steroid injections in thumb base osteoarthritis has been questioned [8], particularly due to the short effect [9] and concern that intra-articular steroids may degenerate articular cartilage in osteoarthritis [10].

Intra-articular injection of platelet-rich plasma (PRP) has arisen as an alternative treatment for osteoarthritis. PRP is derived by centrifugation of autologous blood to separate the plasma containing high platelet concentrations. It is prepared and administrated in the outpatient clinic. PRP is a cocktail of platelets, growth factors, and inflammatory mediators that may be effective for cartilage repair [11]. In addition, the fibrinogen in PRP may be activated to form a fibrin matrix to fill cartilage lesions [12].

PRP has demonstrated effective pain relief and functional improvement compared to hyaluronic acid or saline without increased risk of adverse events in the treatment of knee osteoarthritis [13]. In patients with knee osteoarthritis, PRP is reported to decrease pain more and for a longer duration than steroid injections [14]. There is little data on the effect of PRP on hand osteoarthritis. In a small study of 33 patients, PRP had reportedly better long-standing pain reduction in thumb-base osteoarthritis than intra-articular steroid injections [15]. In another small RCT with short-term follow-up, the effect of PRP compared to corticosteroid and hyaluronic acid showed a positive effect of PRP only at 4 weeks; at 12 weeks, the effect had ceased [16]. Other studies on PRP in the thumb base are retrospective and/or include other joints and therefore have a low level of evidence, and results are contradictory [17, 18]. A recently published meta-analysis concluded the need for a good placebo controlled RCT on PRP in thumb base osteoarthritis [19].

Studies have shown that the concentration of platelets is essential for the effect of PRP in knee osteoarthritis, with higher concentrations providing better chondro-protection and alleviation of symptoms [20], and that repeated injections can result in a better effect [21]. Using a double-spin technique in producing the PRP, the concentration of platelets is even higher than in the single-spin PRP previously most commonly used in intra-articular injections. The thumb base joint is much smaller than the knee, and only a small amount of PRP (< 1 ml) can be injected. Hence, high-concentration PRP may be beneficial in this small joint.

This study aims to evaluate whether a single high-concentration PRP injection decreases pain and disability in patients with thumb base osteoarthritis in the short term and compare the effect to placebo.

### Research questions

Does high-concentration PRP decrease pain and improve function more in the short term than saline injection (placebo) for thumb-base osteoarthritis?

### Hypotheses

1. High-concentration PRP injection will decrease osteoarthritis pain in the thumb base
2. High-concentration PRP injection will decrease pain and improve function more than saline (placebo) for osteoarthritis in the thumb base

## Methods

### Design

**Fig. 1:**
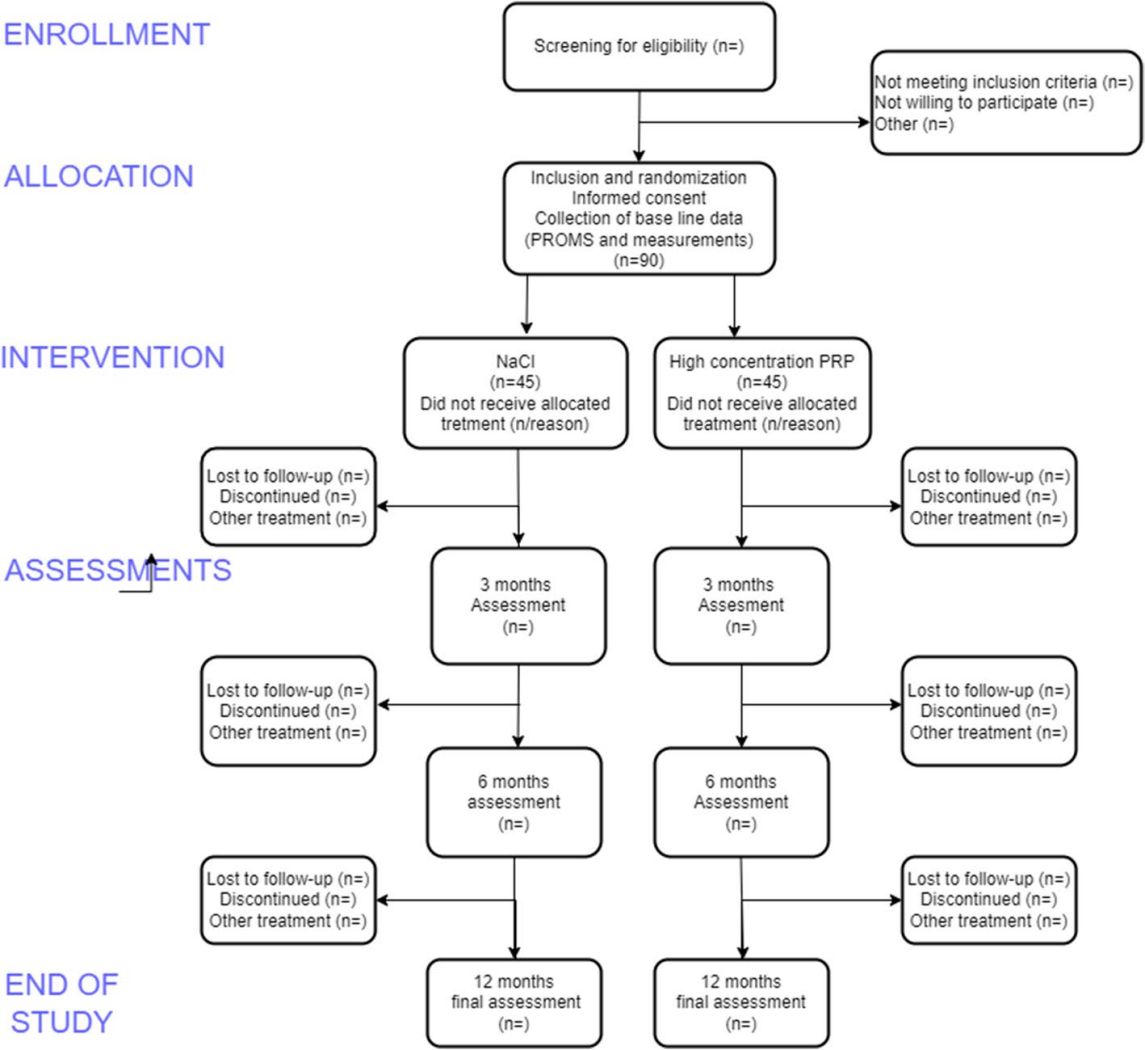
Flowchart of study

**Fig. 2:**
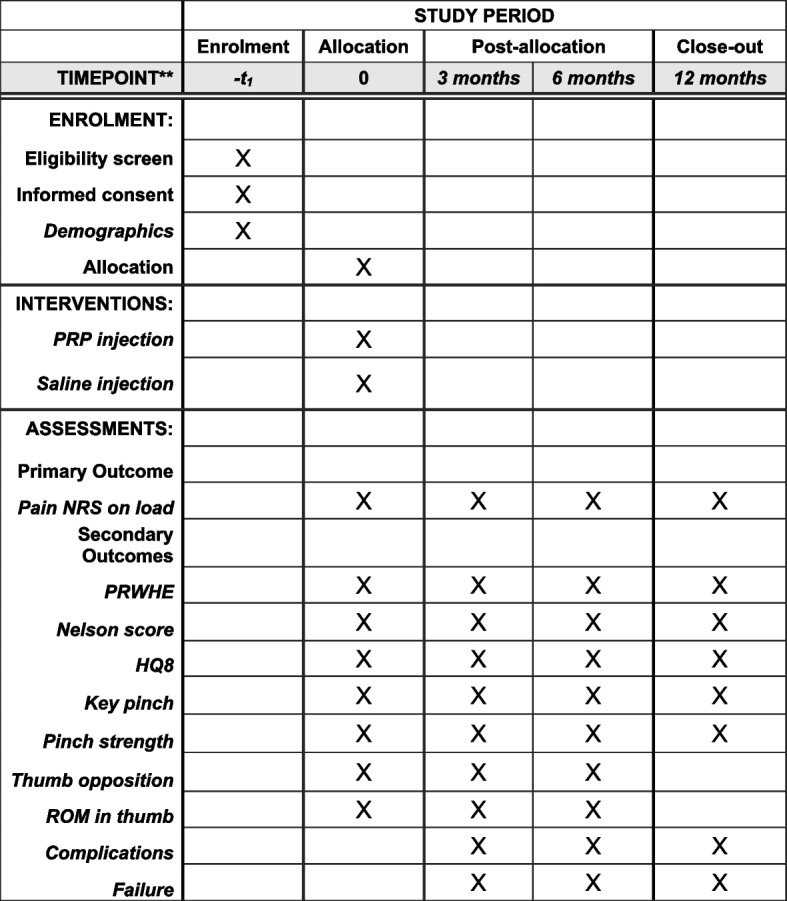
SPIRIT figure

The design is a single-center randomized patient and assessor-blinded controlled trial. Figure 1 shows a flowchart of the study (Fig. 1), and details are listed in the SPIRIT figure (Fig. 2). The SPIRIT checklist is available as a supplement (S1). The study is conducted at the Department of Hand Surgery, Södersjukhuset, a hand trauma unit in Stockholm, with a catchment area of 2.8 million people.

### Randomization

Randomization through sealed envelopes, in batches of 10, is performed by a research nurse. Stratification is used according to radiological Eaton-Littler class (1, 2, and 3). All patients are included by the authors JvK and MW or by three informed colleagues: doctors JS, MM, and KE.

### Interventions

1. High-concentration PRP injection PRP injection (0.6–1 ml). Arthrex ACPmax system
2. Saline injection (0.6–1 ml)

All injections are administered under sterile conditions and radiological guidance on the day of randomization. Injections are performed from the dorsal, just radial, or ulnar of APL and EPB. To mimic the PRP procedure, a blood sample is taken from patients allocated to saline injection, and they must wait in the waiting area for the injection just as long as patients allocated to PRP. Patients wear a blindfold during injection. After the injections, patients do not receive any specific conservative treatment (orthoses, joint protection education). In bilateral thumb base osteoarthritis cases, the more symptomatic joint is injected and assessed.

### Inclusion criteria

The following are the inclusion criteria: osteoarthritis in the thumb base, radiological Eaton-Littler class 1–3 [22], clinical signs of thumb base osteoarthritis (pain at palpation of the CMC-1 joint and pain during the provocation test).

### Exclusion criteria

The following are the exclusion criteria: rheumatoid arthritis diagnosis, ongoing infection in the hand or wrist, history of gout or pseudogout in the hand, inability to co-operate with the follow-up protocol (language difficulties, severe psychiatric disorder, cognitive impairment, drug addiction), chronic pain syndrome/centralized pain, intra-articular injection in the affected joint within 6 months prior to inclusion, Eaton-Littler class 4 (defined as symptomatic STT joint osteoarthritis).

### Outcome

The primary outcome is as follows: change of pain on load (numerical rating scale (NRS) 0–100) in the thumb from before the first injection to 6 months after.

The secondary outcomes are as follows:

- Change in PROMs (Patient-Reported Outcome Measures) from pre-injection to 3, 6, and 12 months, including Patient-Rated Wrist/Hand Evaluation (PRWHE) score [23], Nelson score [24], and HQ-8 score [25]
- Objective physical variables: key pinch and three-finger pinch strength measured by using the Saehan Medical® mechanical pinch gauges SH5010 (the mean value of three consecutive measurements is used), radial and palmar abduction of the first metacarpal [26], thumb opposition [27] (Kapandji score). All measurements are performed according to the manual for measuring motion and strength in the Swedish national quality registry HAKIR [26]
- Time for the patient to experience the need for an intervention in the form of renewed injection, steroid injection, or surgery within 1 year after injection. Any adverse events, complications, and failures (defined as lack of improvement or deterioration of pain) during the study period will be recorded

### Sample size

To show a difference of 20 points (out of 100), representing the minimal clinical difference [28], in pain on load NRS score between PRP and saline injection (SD 30) (3) after 6 months, 37 patients are required in each treatment arm. The power will be 80% (*p* < 0.05). To account for non-parametric outcomes and loss to follow-up, we aim to include 90 patients in total, 45 in each treatment arm.

### Recruitment

Patients diagnosed with painful osteoarthritis in the thumb base at the Department of Hand Surgery at Södersjukhuset, Stockholm, are asked to participate in the study.

### Assessment

- The Patient-Reported Outcome Measure (PROM) questionnaires at baseline, 3, 6, and 12 months after the first injection and Pain Catastrophizing Scale Questionnaire (PCS [29]) at baseline (to be included as a covariate in the analysis)
- Objective physical variables (key and pinch grip, MC-1 radial and palmar abduction, opposition): at baseline 3, 6, and 12 months after injection

One doctor performs the preoperative assessment and injection. A second doctor, blinded to the injection type, performs the assessments at 3 and 6 months. Adverse events are reported. At 6 months, treatment is revealed, and blinding is tested by asking the patients and assessor what treatment they believe the patient has received before disclosure. The assessment at 12 months is not blinded. If patients have persisting pain and significant problems at this point, they can proceed with another line of treatment.

### Statistical analyses

For comparing PRP versus placebo, the Wilcoxon signed rank and rank sum tests and chi-square tests will be used for non-parametric data (change in PROMS and non-normally distributed outcomes at different times). The *T*-test will be used for changes in continuous data (objective physical variables) with a normal distribution. Shapiro–Wilk test will be used for establishing data normality. Generalized estimating equations (GEE) will be used to analyze the effect of injection, adjusting for pain catastrophizing, gender, and Eaton class. A Kaplan Meier analysis of the need for another intervention within 1 year will be formed and continuously recorded.

### Ethics and data management

The study has been approved by the Swedish Ethical Review Authority, and in accordance with this permit, patients are allowed to withdraw their participation in the study without any reason and without consequence for future care. All participants will fill in an informed consent and have contact information to the research nurse and responsible surgeons should any questions or complications occur. All data are stored according to the ethical permit and is regulated under the GDPR. Data are handled in a pseudonymized form and will be presented on group level. The key to the pseudonyms is stored in a secure location at Södersjukhuset and Karolinska Institutet, only available to the authors. Patients’ insurance is covered by the Swedish Patientförsäkringen.

If patients are unable to attend any follow-up visits, they will be asked to fill out the PROMS online.

Results from the study will be published in a scientific journal and presented at hand surgery meetings.

## Discussion

Osteoarthritis in the base of the thumb is a very common condition. The health of the elderly population is steadily improving. Older people need to remain employable and be self-sufficient well into later life. Hence, this condition warrants further research. There is a lack of scientific evidence for many aspects of the treatment of thumb arthritis, and there is a noticeable need to improve and evaluate treatment outcomes. The James Lind Alliance has identified arthritis in the hand and wrist as one of the 10 most important areas within musculoskeletal diseases to research [30].

In meta-analyses and reviews on osteoarthritis and PRP, the recurrent conclusion is the need for large-size RCTs on this subject [7, 19, 31]. Studies on the effect of PRP can be hard to compare due to a variety of factors—the number of injections, the preparation of the PRP (single or double-spin, centrifugation time, etc.), different joints studied, different severity of the osteoarthritis, different treatments in control groups, etc. In this pragmatic trial that reflects the clinical setting in which PRP injections for thumb base osteoarthritis would be employed, we have chosen to use a commercial kit for extracting the PRP. Hence, we will not know the exact concentration of PRP for each patient. As an autologous blood-derived product, the PRP will differ for every patient.

We compare one dose of high-concentrated PRP (double-spin preparation) with a placebo (saline). The theory is that one injection of high-concentrated PRP is as effective as multiple regular PRP injections, which has been reported to be more effective than a single dose of the regular, single-spin, PRP [32] which is why we have chosen the high-concentration PRP for this study.

We stratify the randomization according to the radiological severity of the disease since previous literature supports a better effect of in the early stages of the osteoarthritis in hand and foot [31].

The sample size is estimated based on the primary outcome, pain on load measured in NRS, which is an established outcome measure in research on musculoskeletal pain [33]. We used the pain on load score in the HQ-8, a questionnaire developed for the Swedish hand surgery quality registry [25]. Pain NRS is a tool that is highly sensitive for individual perception of pain. Therefore, we will adjust the analysis for pain catastrophizing. We have also increased the sample size somewhat to consider the uncertainty of and expected variance of PROMs as outcomes.

The follow-up period is relatively short. The rationale for the primary outcome after 6 months is that with time the risk will increase that the patients require further interventions such as injections or even surgery during the study period. All patients will be assessed after 12 months, but some will have had other treatments by then that may affect the results. Hence, this study will not show long-term effects of PRP. A study with a longer follow-up period to see long-term effect of PRP could be a future perspective if we find PRP effective in the short term.

This is a single-center study. A future improvement could be to include more centers or other osteoarthritic joints to establish that the results are valid also in diverse patient populations.

The foremost strengths of this trial are the size and the randomized double-blinded design.

## Trial status

This is protocol version 1 dated November 21, 2023. Ethical approval was obtained December 11, 2023. The first patient was included January 17, 2024. Predicted final inclusion during the second half of 2025 which entails data ready for analysis in the autumn of 2026.

## Supplementary Information

Additional file 1: S1. SPIRIT Checklist

## Data Availability

Data in this study is restricted by the General Data Protection Regulation (GDPR) (https://www.imy.se/en/organisations/data-protection/). Data can be made be available after pseudonymization for researchers after application to and approval by the Swedish Ethical Review Authority and the local data safety committee at Södersjukhuset (GDPR.sodersjukhuset@regionstockholm.se).

## Abbreviations

CMC-1: The first carpometacarpal joint
PRP: Platelet-rich plasma
APL: Abductor pollicis longus
EPB: Extensor pollicis brevis
STT: Scaphotrapeziotrapezoidal joint
NRS: Numerical rating scale
PROM: Patient-Reported Outcome Measure
PRWHE: Patient-Rated Wrist/Hand Evaluation
HQ-8: HAKIR eight-item participant questionnaire
GEE: Generalized estimating equations
RCT: Randomized controlled study
GDPR: General Data Protection Regulation
SPIRIT: Standard Protocol Items: Recommendations for Interventional Trials
